# Ferrets as a model for investigating the impact of chemical agents on cerebral cortical sulcogyrogenesis

**DOI:** 10.3389/fneur.2026.1796867

**Published:** 2026-05-11

**Authors:** Kazuhiko Sawada

**Affiliations:** Department of Nutrition, Faculty of Medical and Health Sciences, Tsukuba International University, Tsuchiura, Japan

**Keywords:** ferret, gyrification, gyrus, LPS, neurogenesis, subventricular zone, sulcus, VPA

## Abstract

Gyrencephalic features of the cerebral cortex have been documented in many mammals, exhibiting unique species-related patterns, and are disturbed in individuals with neurodevelopmental and/or psychiatric disorders, including autism spectrum disorder. This mini-review article provides an overview of a series of studies that have investigated sulcogyral developmental alteration as the outcome of two chemical agents with the potential to cause autism spectrum disorder, valproic acid (VPA) and lipopolysaccharide (LPS), using ferrets, which are gyrencephalic animals. Ferret pups received an injection of VPA (200 μg/g body weight) or LPS (500 μg/g of body weight) at 6 and 7 days of postnatal ages. VPA acts on the subventricular zone progenitors, including basal radial glia, to promote their differentiative division, resulting in shallow sulcal infolding in the rostrovental and medial cortices, while enhancing sulcal infolding in the dorsolateral cortex. In contrast, the apoptosis of cortical neurons derived from postproliferative subventricular zone progenitors in neonatally LPS-exposed ferrets may be involved in an anterior shift of the primary sulci located on the medial and dorsolateral cortices. These two agents affect subventricular zone progenitors differently, leading to distinct gyrification abnormality patterns. These findings suggest that the ferret is an advantageous animal model for elucidating the pathogenesis of neurodevelopmental and/or psychiatric disorders in terms of gyrification abnormalities raised by chemical agents.

## 1 Introduction

Many mammalian species have a folded surface morphology of the cerebral cortex, forming species-related unique patterns of cerebral sulci and gyri (1) that create a topographic partition related to functionally distinct cortical regions (2). Cerebral sulci and gyri emerge in a regular order during the second-trimester equivalent in primates (3, 4) and modify their morphology after the third-trimester equivalent with the local expansion of the cortex and determination of sex, left/right side, and interindividual differences (5–7). Gyrification abnormalities, referred to as dysforming cerebral sulci and gyri, have been documented in humans with neurodevelopmental and psychiatric disorders, such as autism spectrum disorder (ASD), attention-deficit hyperactivity disorder (8) and schizophrenia (9).

Ferrets are carnivorous laboratory animals that have gyrencephalic morphology of the cerebral cortex. This animal undergoes neurodevelopmental events, such as sulcogyrogenesis and the late stage of corticoneurogenesis, during the first 2 weeks of postnatal life, which occur *in utero* in primates (10–12). This characteristic allows for the direct application of experimental manipulations, such as drug administration, to ferret pups during cortical neurogenesis associated with sulcogyrogenesis. This mini-review describes our research on altered sulcogyrogenesis induced by neonatal exposure to two different chemical agents that can potentially cause ASD, valproic acid (VPA) and lipopolysaccaride (LPS) (13, 14).

## 2 Sulcal and gyral development

Sulcal and gyral development has been reported in the ferret cortex, as defined by gross anatomical (12) and *ex vivo* magnetic resonance imaging (MRI) (11) studies. The chronological sequence of sulcal and gyral emergences was reproducible between these two approaches and was briefly mapped on an illustration of the lateral surface of the left cerebral hemisphere as shown in Supplementary Figure S1. Sulcogyrogenesis of the ferret cortex begins with the emergence of the rostral suprasylvian sulcus (rsss), crucial sulcus (crs), and rhinal fissure (rf) as shallow grooves on the lateral surface by postnatal day (PD) 4. The rostrovental and medial cortical regions corresponding to the motor, somatosensory, and cingulate cortices form the splenial sulcus (ss), presylvian sulcus (prs), coronal sulcus (cns), and pseudosylvian sulcus (pss) during PDs 4 and 10, respectively. These sulci demarcate the anteiror sigmoid gyrus (ASG), posterior sigmoid gyrus (PSG), coronal gyrus (CNG) and anterior ectosylvian gyrus (AEG). The caudodorsal cortical region corresponding to the posterior parietal, auditory, and visual cortices is infolded to delineate the lateral sulcus (ls), and the caudal suprasylvian sulcus (csss) emerges during PDs 10 and 21. These sulci demarcate the lateral gyrus (LG), suprasylvian gyrus (SSG), posterior ectosylvian gurus (PEG) and visual cortical area (VCA).

## 3 Effect of neonatal exposure to VPA

**Figure 1:**
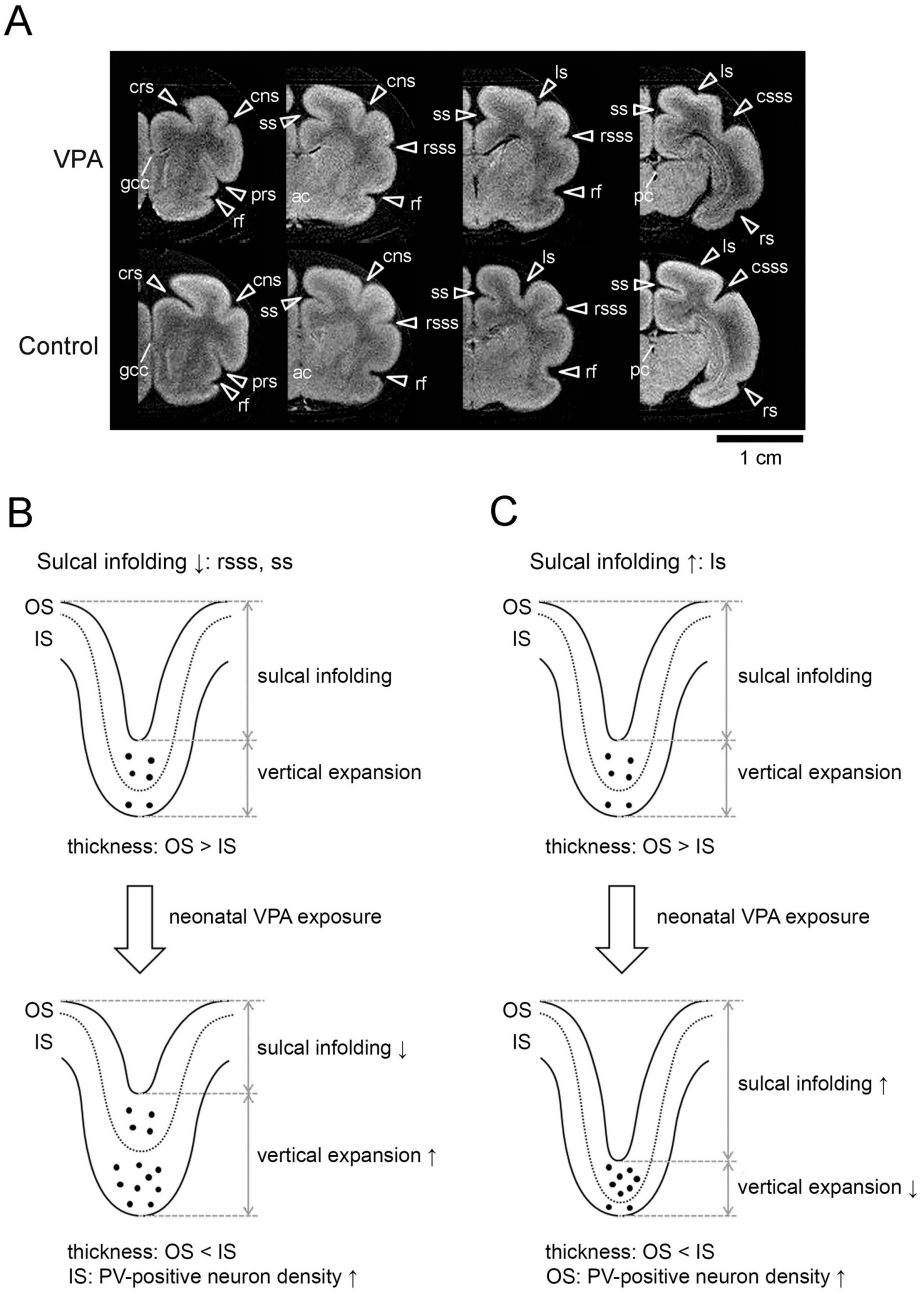
Three dimensional anatomical MR images of the cerebrum and changes in sulcal infolding based on the mechanical hypothesis in neonatally VPA-exposed ferrets. **(A)** *Ex vivo* cerebrum MRIs in the coronal plane generated using RARE sequence with short TR and minimum TE settings. Coronal MRIs are shown from the left to right in the following order: the prefrontal region at the rostral end of the genu of the corpus callosum (gcc), the frontal region at the anterior commissure (ac), the parietotemporal region at the caudal end of the rhinal fissure (rf), and the parieto-occipital region at the posterior commissure (pc). **(B)** Neonatal exposure to VPA enhances the density of parvalbumin (PV)-positive neurons and thickened the inner stratum (IS) of the rostral suprasylvian sulcus (rsss) and splenial sulcus (ss) floors. This may enhance vertical expansion, thereby reducing sulcal infolding. **(C)** Neonatal exposure to VPA enhances the density of PV-positive neurons and thickened the outer stratum (OS) of the lateral sulcus (ls) floor. This may reduce vertical expansion, thereby enhancing sulcal infolding. Bar = 1 cm. as, ancinate sulcus; cns, coronal sulcus; crs, cruciate sulcus; csss, caudal suprasylvian; ls, lateral sulcus; prs, presylvian sulcus; rs, rhinal sulcus; rsss, rostral suprasylvian sulcus; ss, splenial sulcus. Adapted from Figure 2C, 8B, and 8C of Sawada et al. (13).

VPA is an antiepileptic/anticonvulsant drug that also acts as an epigenetic regulator by inhibiting histone deacetylases 1 and 2 (15, 16). Prenatal and neonatal exposure to VPA alters brain development, resulting in ASD-like behaviors, including impaired social interactions in rodents (17) and ferrets (18). Here, the findings of our investigation on the effect of neonatal VPA exposure on sulcogyrogenesis in ferrets (13) are briefly described as follows. Ferret neonates were given an injection of VPA at 200 μg/g of body weight on PDs 6 and 7, corresponding to the late stage of cortical neurogenesis. At PD 20, when completing primary sulogyrogenesis, sulcal infolding was reduced in the ss and rsss by the thickening of sulcal floors, but enhanced in the ls by the thinning of sulcal floors, in VPA-exposed ferrets (Figure 1A). These changes were attributed to an increased density of neurons, which transiently express parvalbumin, in the inner cortical stratum of the ss and rsss floors and in the outer cortical stratum of ls floors (Figures 1B,C), both of which are involved in VPA-promoted proliferation of subventricular zone (SVZ) progenitors including basal radial glia (bRG) followed by their neural differentiation (differentiative division) in P7 ferret neonates (19).

## 4 Effect of neonatal exposure to LPS

**Figure 2:**
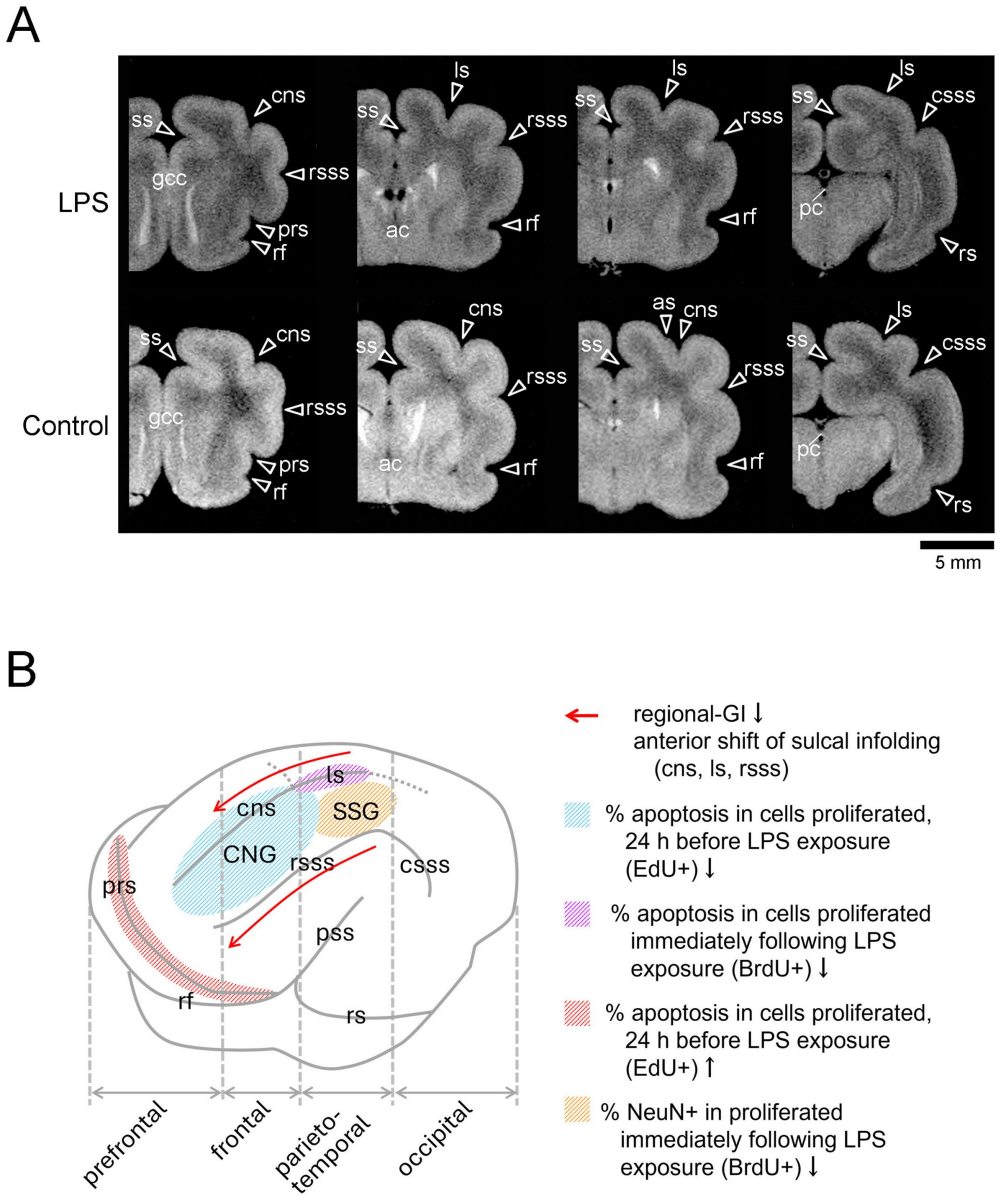
Three-dimensional anatomical MR images of the cerebrum and summary of the MRI-based morphometric and quantitative immunohistochemical findings in LPS-exposed ferrets. **(A)** *Ex vivo* cerebrum MRIs in the coronal plane generated using RARE sequence with short TR and minimum TE settings. Coronal MRIs are shown from left to right in the following order: the prefrontal region at the rostral end of the genu of the corpus callosum (gcc), the frontal region at the anterior commissure (ac), the parietotemporal region at the caudal end of the rhinal fissure (rf), and the parieto-occipital region at the posterior commissure (pc). **(B)** Illustration of changes in sulcal infolding and apoptosis of cortical neurons by neonatal LPS exposure. Bar = 5 mm, as, ancinate sulcus; cns, coronal sulcus; crs, cruciate sulcus; csss, caudal suprasylvian; ls, lateral sulcus; prs, presylvian sulcus; rs, rhinal sulcus; rsss, rostral suprasylvian sulcus; ss, splenial sulcus. Adapted from Figure 3C and 12 of Sawada et al. (14).

LPS is an endotoxin of gram-negative bacteria and activates the innate immunity, as do peptidoglycan, *β*-glycan, and polyinosinic:polycytidylic acid (Poly I: C). LPS is often used to generate an animal model of maternal immune activation (MIA) that mediates microglial activation in dams to secrete pro-inflammatory and anti-inflammatory cytokines, increasing the risk of neurodevelopmental disorders including ASD in offsprings (20, 21). Besides, LPS acts directly on neural stem/progenitor cells via the Toll-like receptor 4 (TLR4) to regulate their proliferation and differentiation (22). We investigated the effect of neonatal LPS exposure on sulcogyrogenesis using ferrets (14) and briefly describe the findings as follows. Ferret neonates were given an injection of LPS at 500 μg/g of body weight on PDs 6 and 7 to investigate the direct LPS effects on SVZ progenitors, independent of MIA. At PD 20, when primary sulogyrogenesis was completed, sulcal infolding was shifted anteriorly in the primary sulci located on the medial and dorsolateral cortices, such as the ss, cns, rsss, and ls in LPS-exposed ferrets (Figures 2A,B). These changes may be involved in region-related modulation of apoptosis of cortical neurons, which were derived from postproliferative SVZ progenitors following LPS exposure: apoptosis is enhanced in the prs and ls floors located on the prefrontal and parietal association cortical regions; and apoptosis is reduced in the cns floors and the CNG crowns located on the primary motor cortex (Figures 2A,B). Although LPS promotes the differentiative division of SVZ progenitors, mainly intermediate progenitors (IPs) (23), this may not be essential for altered sulcal infolding in LPS-exposed ferrets.

## 5 Conclusion

Gyrification is the process of creating sulci and gyri during the morphogenesis of the cerebral cortex and is altered by genetic and environmental factors (3). Manipulation of genes regulating cortical neurogenesis in SVZ progenitors including IPs and bRG causes gyrification abnormalities in ferrets (24). Our findings described in this mini-review quantitatively investigated gyrification abnormalities triggered by exposure to two different chemical agents, VPA and LPS, both of which have the potential to cause ASD by targeting the late stage of cortical neurogenesis. VPA mainly acts on the bRG (19), and LPS mainly acts on IPs (23). Furthermore, the gyrification abnormalities caused by these two agents were distinct. Thus, the types of gyrification abnormalities caused by specific chemical agents could be identified in ferrets. These findings suggest that ferrets are advantageous animal models for elucidating the pathogenesis of neurodevelopmental and/or psychiatric disorders in terms of gyrification abnormalities caused by chemical agents. In addition, early postnatal age in ferrets corresponds to third trimester stage of human brain development. Many studies have administered chemical agents, including VPA and LPS during the mid-pregnancy, corresponding to the second trimester, to establish models of ASD and other neurodevelopmental disorders in lissencephalic rodents (18, 25–30). Therefore, behavioral defects observed in ferret models with gyrencephalic abnormalities should be compared with findings from rodent models, including documented MIA models, only with caution. This limitation is crucial when using our ferret model in research on neurodevelopmental disorders.
