# Supplementary material for: Ferrets as a model for investigating the impact of chemical agents on cerebral cortical sulcogyrogenesis
Source: Front Neurol. 2026 May 11;17:1796867. doi: 10.3389/fneur.2026.1796867 (PMC13199045; doi:10.3389/fneur.2026.1796867)
Supplement: Supplementary file 1 [file Table_1.docx]

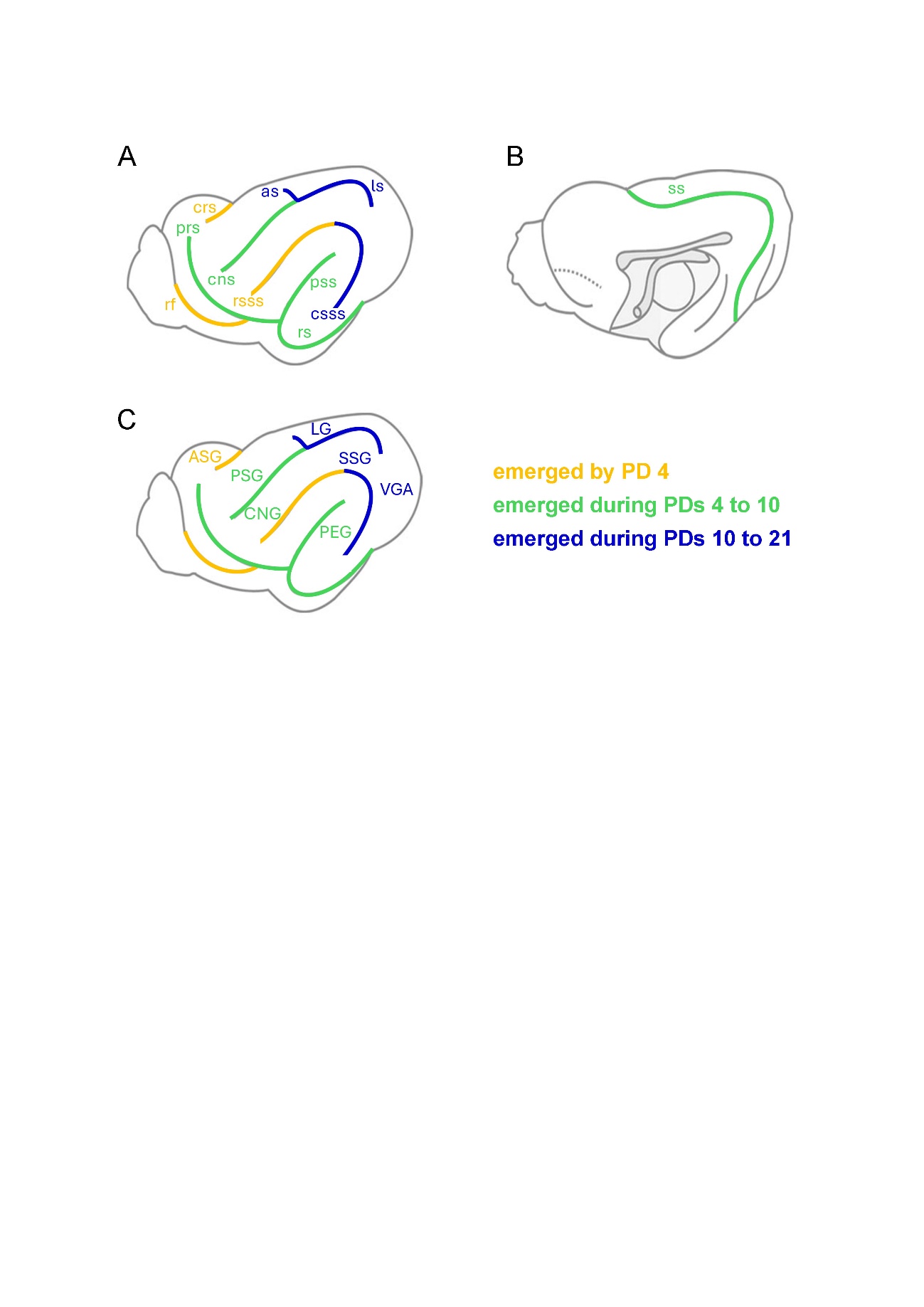


**Figure S1.** Summaries of sulcal and gyral emergences of ferret cerebrum. A. Chronology of sulcal emergence on the lateral surface of cerenral cortex coded by colors. B. Chronology of sulcal emergence on the medial surface of cerenral cortex coded by colors. C. Chronology of gyral emergence on the lateral surface of cerenral cortex coded by colors. Orange color indicates sulci or gyri emergence on postnatal day (PD) 4; green color indicates sulci or gyri emergence during PDs 4 to 10; blue color indicates sulci or gyri emergence during PDs 10 to 21. as, ancinate sulcus; cns, coronal sulcus; cruciate sulcus; csss, caudal suprasylvian sulcus; ls, lateral sulcus; prs, presylvian sulcus; rf, rhinal fissure; rs, rhinal sulcus; rsss, rostral suprasylvian sulcus; ss, splenial sulcus.
